# 

**Published:** 1867-09

**Authors:** 


### CONTENTS FOR SEPTEMBER.

ORIGINAL CONTRIBUTIONS.

Propolis as a remedy for Diarrhœas, Acute and Chronic. By H. O. Hitchcock, M.D., Kalamazoo, Mich. ---- 417

On the improved method of treatment of Diseases of the Lungs, Throat, and Nasal Passages, by Inhalations of Atomized Fluids. By Wm. C. Lyman, M.D., late Surgeon U. S. Navy, Resident Physician U. S. Marine Hospital, etc. ---- 420

Nasal Polypous. By R. P. Hunt, M.D., of Chicago. ---- 429

The Endoscope, and its Application to the Diagnosis and Treatment of Urinary Affections. By A. J. Desormeaux, M D., Translated by R. P. Hunt, M.D. Continued from Page 400 ---- 449

PROCEEDINGS OF SOCIETIES.

Chicago Medical Society.—Shell wound of the Head. ---- 432

Cancerous Disease of the Œsophoagus, with Ulceration into the Bronchi. ---- 433

Encephaloid mass penetrating the right Renal vein and the Vena Cava. ---- 438

Curvature of the Spine. ---- 434

Successful removal of an Ovarian Tumor. ---- 434

Tumor of the Choroid. ---- 435

Peoria County Medical Society. ---- 435

FOREIGN CORRESPONDENCE. ---- 436 and 439

EDITORIAL.

Meeting of the Alumni of Rush Medical College. ---- 443

Women-Doctors. ---- 443

To Subscribers. ---- 444

Cincinnati Changes. ---- 444

George K. Amerman, M.D. ---- 444

Erratum. ---- 446

Crowded Out. ---- 446

Books and Pamphlets Received ---- 446

